# Supplementary material for: Prior flavivirus immunity skews the yellow fever vaccine response to cross-reactive antibodies with potential to enhance dengue virus infection
Source: Nat Commun. 2024 Feb 24;15:1696. doi: 10.1038/s41467-024-45806-x (PMC10894228; doi:10.1038/s41467-024-45806-x)
Supplement: Supplementary file 1 — Supplementary Information [file 41467_2024_45806_MOESM1_ESM.pdf]

	Participants of the main study n = 250	TBEV-vaccinated n = 139	TBEV-unvaccinated n = 56
**Year of study inclusion**			
2015	32	21	5
2016	69	38	18
2017	83	47	18
2018	52	30	13
2019	14	3	2
**Sex**			
Female	169	99	38
Male	81	40	18
**Median age in years (range)**	24 (19-47)	24 (20-46)	24.5 (21-38)
**Median weight (kg)**	65	64	63
**Body Mass Index (BMI)**			
Underweight (≤ 18.49 kg/m2)	8	3	4
Normal weight (18.50–24.99 kg/m2)	210	121	48
Overweight (25.00–29.99 kg/m2)	27	12	4
Obesity (≥ 30.00 kg/m2)	3	2	0
Missing	2	1	0
**Travel to flavivirus-endemic areas**			
Yes	24	12	8
No	226	127	48
**Ethnicity:**			
European	242	55	133
East Asian	3	0	3
Native American	2	0	2
NA	3	1	1
**Self-reported infection two weeks prior to vaccination**	45	26	11

P value across TBEV-vaccinated and unvaccinated >0.05 in all cases (chi-squared test)

Supplementary Table 1. Cohort-1 characteristics

Supplementary Figure 1

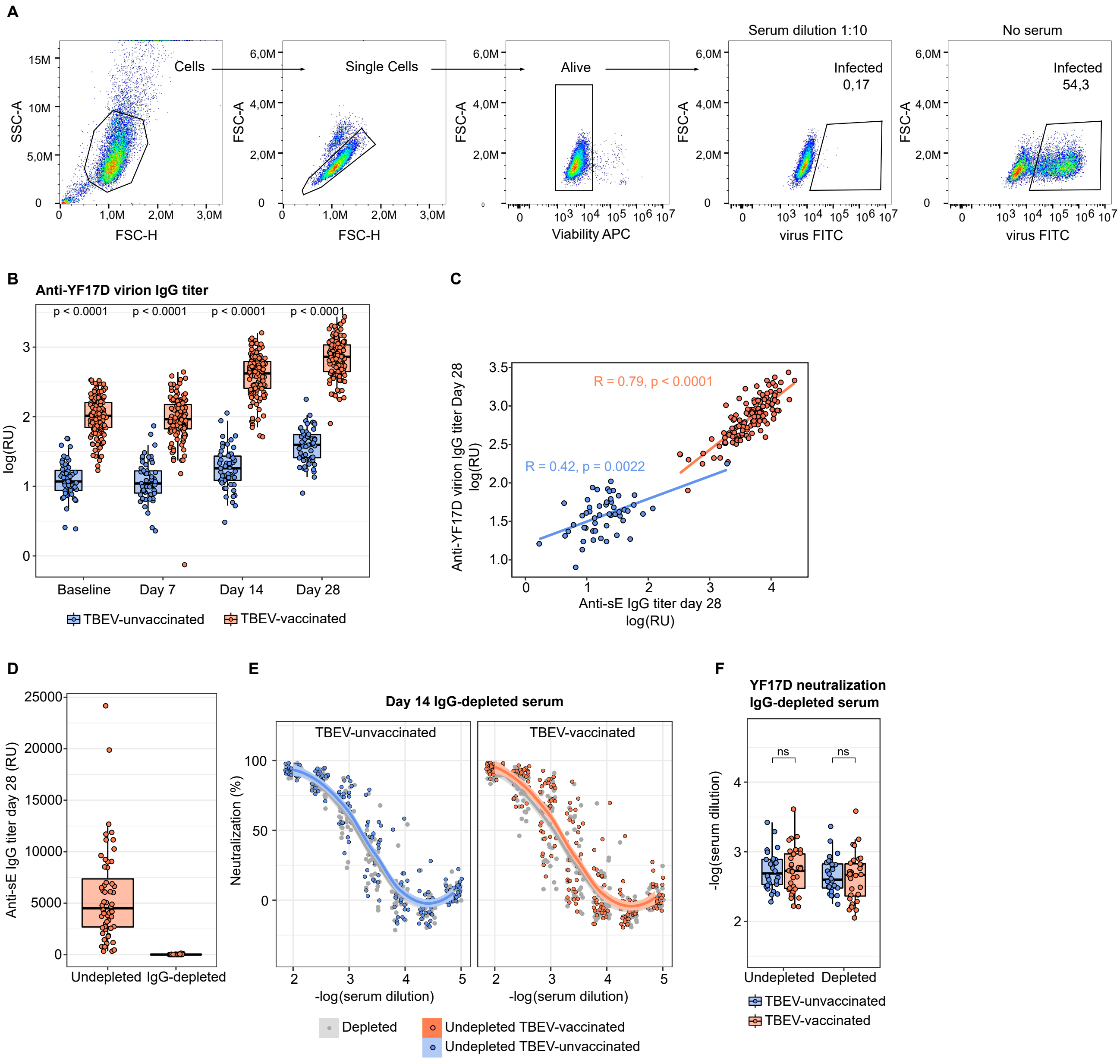

Supplementary Figure 1. YF17D-induced antibody response.

A) Representative gating strategy for the FluoRNT assay using a neutralizing serum sample. **B)** Longitudinal YF17D virion-specific IgG quantification for TBEV-vaccinated (n = 137) and TBEV-unvaccinated (n = 55) individuals. **C)** Spearman correlation between anti-E IgG and anti-YF17D virion titer on day 28 (n =132 TBEV-vaccinated and n = 52 TBEV-unvaccinated donors). **D)** IgG depletion validation quantified in an anti-E protein ELISA (n = 67). **E)** Neutralization curves of day 14 pv undepleted polyclonal serum and the respective IgG-depleted serum (in grey) for TBEV-pre-vaccinated (n = 32, in orange) or unvaccinated individuals (n = 28, in blue). **F)** Quantification of the 80% neutralization cutoff before and after IgG depletions on day 14 pv shown in E).
Boxplots show a horizontal line indicating the median and lower and upper hinges corresponding to the first and third quartiles. The lower and upper whiskers extend to 1.5xIQR (inter-quartile range) from the respective hinge. The curve fitting in E) was done with local regression with a 0.95 confidence interval. Statistical significance in B) and F) was estimated with a two-sided Mann-Whitney test between TBEV-vaccinated and unvaccinated individuals and is shown above every comparison. P values above 0.05 are considered non-significant (ns).

Supplementary Figure 2

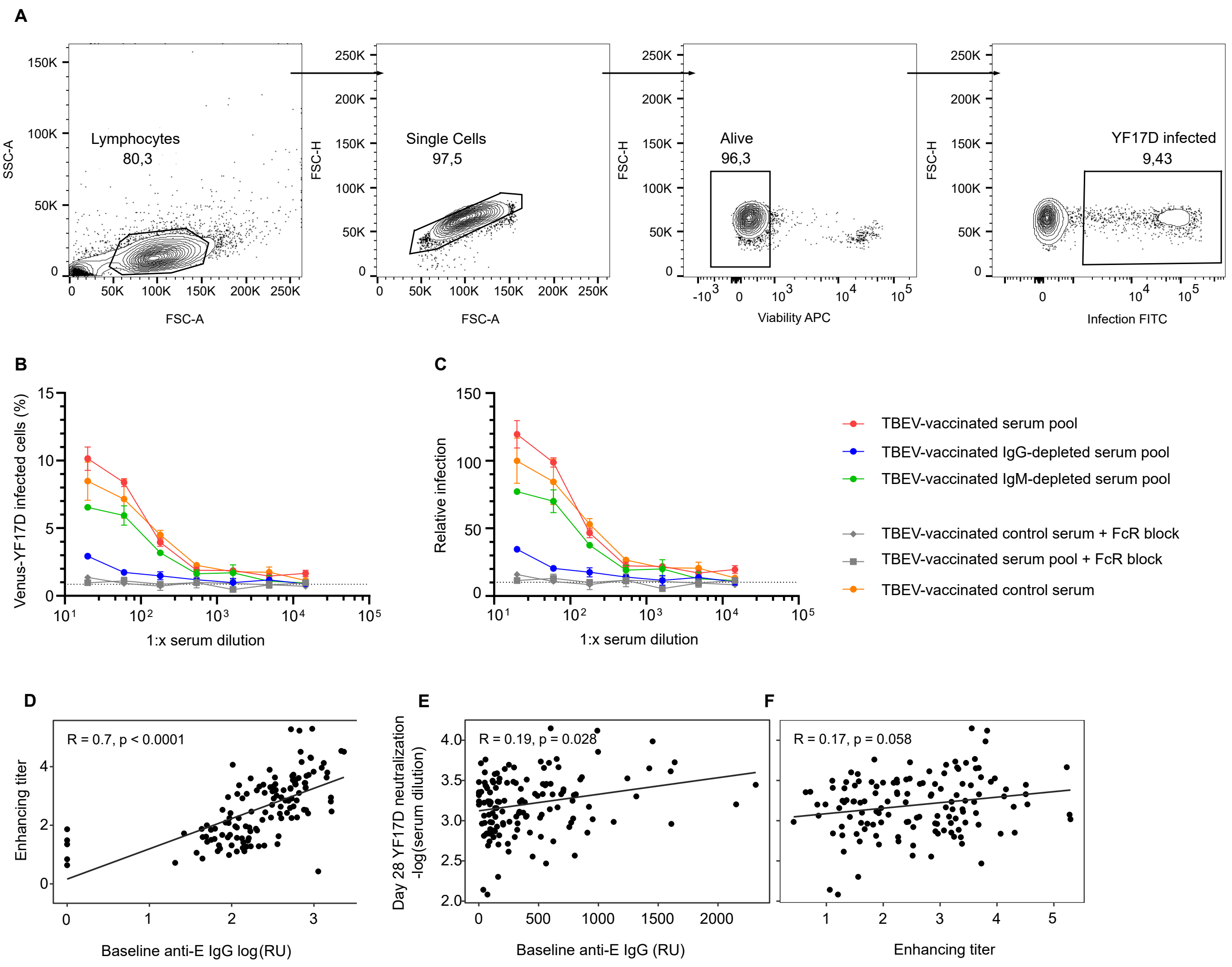

Supplementary Figure 2. Antibody dependent enhancement of YF17D virus infection.

A) Gating strategy for the antibody-dependent enhancement of YF17D-venus infection of THP-1 cells. **B-C)** YF17D ADE on THP1 cells mediated by undepleted serum or serum with anti-FcγR blocking antibodies and serum depleted for IgG or IgM antibodies. Serum pools of TBEV-vaccinated individuals at baseline and the internal control used for normalization across assays were included. Frequency of infected cells (B) was normalised against the internal control as described in methods to obtain the relative infectivity values (C). Error bars indicate the range of technical replicates **D-F)** Spearmann correlations of the enhancing titer with anti-E IgGs at baseline and with the IgG and neutralizing titers following YF17D vaccination (D, n = 130 pairs; E, n = 134 pairs; F, n = 132 pairs of TBEV-vaccinated individuals)

Supplementary Figure 3

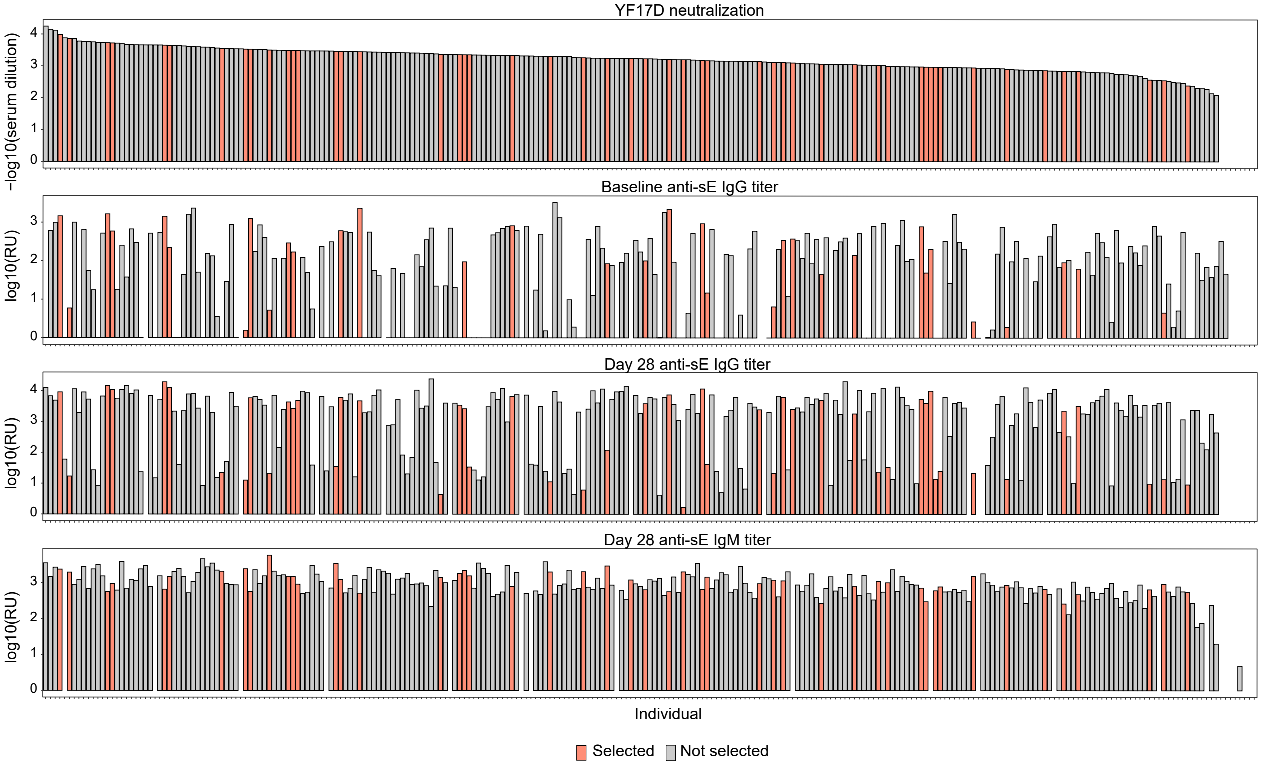

Supplementary Figure 3. Selection of individuals for thorough characterization

Individuals selected for further analysis (in red). Individuals were selected to represent different neutralizing capacities and antibody titers after vaccination. These samples were used in the following assays: cross-reactivity assay, antigen-specific depletions, DENV and ZIKV ADE, ELISpot and end-point IgG titer quantification against the recombinant dimers.

Supplementary Figure 4

A

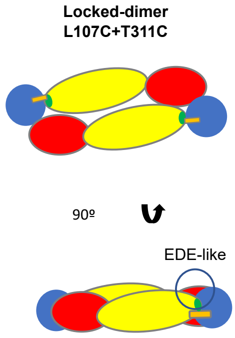

B

Locked-dimer L107C T311C	Expected epitope display	Real epitope display
Epitope specificity	EDE-like, DI, DII, DIII	DI, DIII
Undepleted fraction (remaining specificities)	FLE	EDE-like, FLE, DII

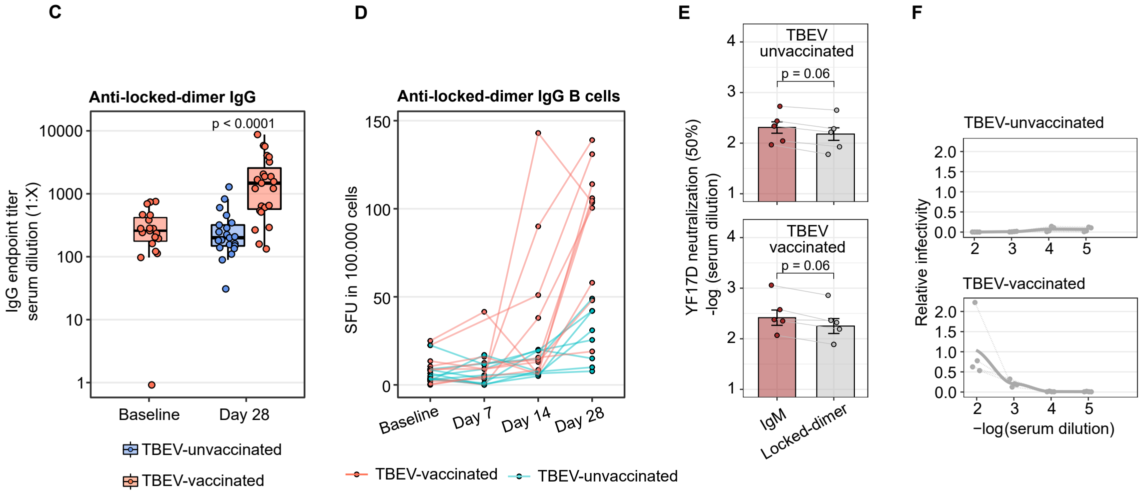

Supplementary Figure 4. Locked-dimer-specific IgG and B cell response.

A) Representation of the recombinantly produced locked-dimer construct with the disulphide bond formed between the mutated L107C and T311C. **B)** Table summarizing the expected epitope display for the locked-dimer construct versus the observed real epitope display. Likewise, the table shows the expected remaining specificities after antigen-specific depletions and the actual undepleted fraction. **C)** Quantification of IgG endpoint titers against locked-dimer protein at baseline (n = 19 TBEV-vaccinated donors) and day 28 (n = 21 TBEV-unvaccinated and n = 26 TBEV-vaccinated individuals). **D)** Longitudinal enumeration of IgG-producing B-cells specific for the locked-dimer antigen (n = 9 TBEV-unvaccinated and n = 10 TBEV-vaccinated individuals). Units represent spot-forming units per 100.000 PBMC. **E)** YF17D neutralization titers (50% cutoff) of IgM-depleted and locked-dimer-depleted sera (n = 5 TBEV-unvaccinated and n = 5 TBEV-vaccinated individuals). **F)** Dengue ADE driven by IgM and locked-dimer-specific IgG-depleted sera n = 4 TBEV-unvaccinated and n = 4 TBEV-vaccinated individuals).

Boxplots show a horizontal line indicating the median and lower and upper hinges corresponding to the first and third quartiles. The lower and upper whiskers extend to 1.5x IQR from the respective hinge. Barplots indicate the mean and error bars the standard error of the mean. Statistical significance between TBEV-vaccinated and unvaccinated individuals (C) was estimated with a two-sided Mann-Whitney test. Statistical significance in F was calculated with a two-sided Wilcoxon signed-rank test. P values above 0.05 are considered non-significant (ns).

Supplementary Figure 5

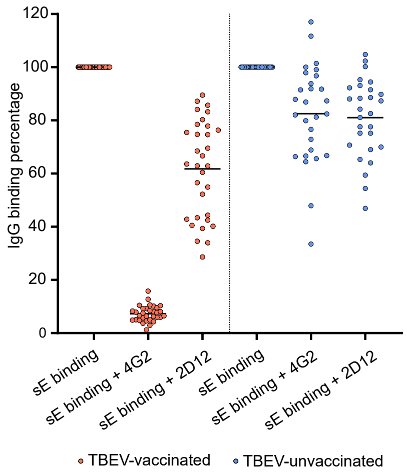

Supplementary Figure 5. 4G2 and 2D12 competition.

IgG competition binding assay to sE in the presence or absence of competing 2D12 or 4G2 monoclonal antibodies. Serum samples from 28 days post YF17D vaccination were used for TBEV-pre-vaccinated (n = 32, in red) and TBEV-unvaccinated (n = 28, in blue) inividuals.

Supplementary Figure 6

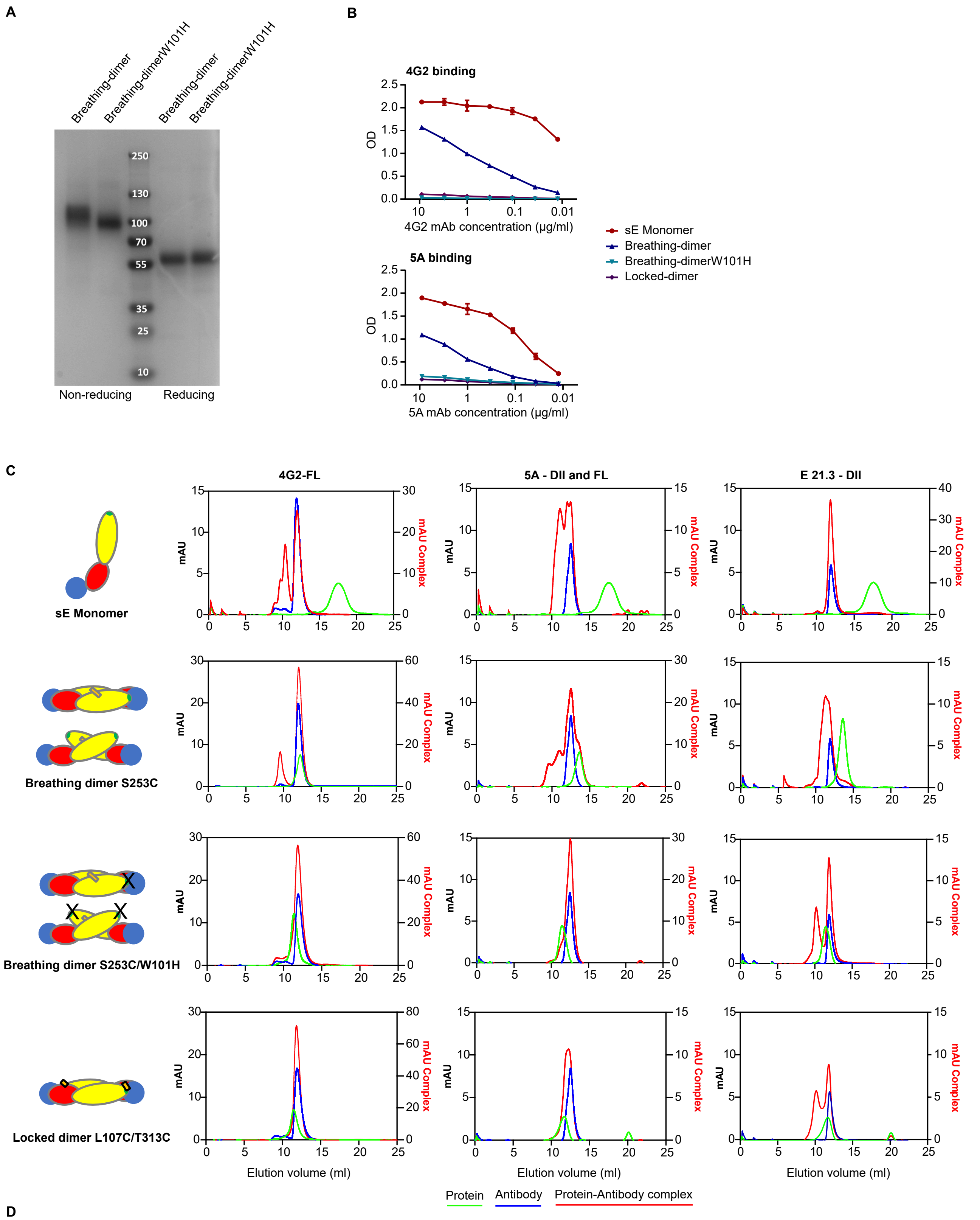

D

YF17D E protein mutants binding to specific antibodies

	4G2 (FLE)	5A (DII/FLE)	E21.3 (DII)
sE Monomer	+	+	+
Breathing dimer	+	+	+
Breathing-dimerW101H	-	-	+
Locked-dimer	-	-	+

Supplementary Figure 6

A) SDS-PAGE of the breathing-dimer and breathing-dimerW101H under non-reducing and reducing condition (+ditiotreitol). Coomassie blue staining. **B)** Binding ELISA of the monoclonal 5A and 4G2 antibodies to the plate-coated sE (red), breathing-dimer (dark blue), breathing-dimer^{W101H} (light blue) and locked dimer (purple) constructs. Error bars indicate the range of technical replicates. **C)** Size exclusion chromatography elution profile of the antibody (blue), protein (green) or antibody-protein complexes (red). The left y-axis indicates the ultraviolet absorbance. **D)** Table summarizing the binding capacity of 5A, 4G2 and E21.3 mAb to the different protein constructs.
