# Supplementary material for: Prior flavivirus immunity skews the yellow fever vaccine response to cross-reactive antibodies with potential to enhance dengue virus infection
Source: Nat Commun. 2024 Feb 24;15:1696. doi: 10.1038/s41467-024-45806-x (PMC10894228; doi:10.1038/s41467-024-45806-x)
Supplement: Supplementary file 3 — Reporting Summary [file 41467_2024_45806_MOESM3_ESM.pdf]

nature portfolio

Corresponding author(s): Simon Rothenfusser

Last updated by author(s): 29/01/24

Reporting Summary

Nature Portfolio wishes to improve the reproducibility of the work that we publish. This form provides structure for consistency and transparency in reporting. For further information on Nature Portfolio policies, see our Editorial Policies and the Editorial Policy Checklist.

Statistics

For all statistical analyses, confirm that the following items are present in the figure legend, table legend, main text, or Methods section.

n/a	Confirmed	
☐	☒	The exact sample size (*n*) for each experimental group/condition, given as a discrete number and unit of measurement
☐	☒	A statement on whether measurements were taken from distinct samples or whether the same sample was measured repeatedly
☐	☒	The statistical test(s) used AND whether they are one- or two-sided<br>*Only common tests should be described solely by name; describe more complex techniques in the Methods section.*
☒	☐	A description of all covariates tested
☐	☒	A description of any assumptions or corrections, such as tests of normality and adjustment for multiple comparisons
☐	☒	A full description of the statistical parameters including central tendency (e.g. means) or other basic estimates (e.g. regression coefficient) AND variation (e.g. standard deviation) or associated estimates of uncertainty (e.g. confidence intervals)
☐	☒	For null hypothesis testing, the test statistic (e.g. *F*, *t*, *r*) with confidence intervals, effect sizes, degrees of freedom and *P* value noted<br>*Give P values as exact values whenever suitable.*
☒	☐	For Bayesian analysis, information on the choice of priors and Markov chain Monte Carlo settings
☒	☐	For hierarchical and complex designs, identification of the appropriate level for tests and full reporting of outcomes
☐	☒	Estimates of effect sizes (e.g. Cohen's *d*, Pearson's *r*), indicating how they were calculated

Our web collection on statistics for biologists contains articles on many of the points above.

Software and code

Policy information about availability of computer code

Data collection

1) BD FACSCanto (sn: V96300312) using BD FACSDiva™ Software and CytoFLEX LX (sn: BE48044) using provided software
2) FLUOstar Omega reader (BMG Labtech) using provided software for ELISA, and luciferase assays.
3) ELISpot Reader ELR04 654 SR (AIDAutoimmun Diagnostika GmbH, Strassberg, Germany) using provided software for spot counting with manual validation
4) Fluorescent microscope (EUROStar 3 PLUS, EUROIMMUN Medizinische 527 Labordiagnostika AG, Lübeck, Germany)
5) No self-made code was used to collect data.

Data analysis

1) Data analysis was done using R version 4. (drc package for dose response analysis, neutralization and end-point titers and ggplot2 package for graphs)
2) Graphpad Prism V8 (GraphPad, La Jolla, CA, 540 USA)
3) FlowJo_v10.8.0 for flow cytometry data analysis
4) Structure figures were created using PyMOL 2.5.2 (Schrédinger, LLC, pymol.org/2/)

For manuscripts utilizing custom algorithms or software that are central to the research but not yet described in published literature, software must be made available to editors and reviewers. We strongly encourage code deposition in a community repository (e.g. GitHub). See the Nature Portfolio guidelines for submitting code & software for further information.

Data

Policy information about availability of data

All manuscripts must include a data availability statement. This statement should provide the following information, where applicable:

- Accession codes, unique identifiers, or web links for publicly available datasets
- A description of any restrictions on data availability
- For clinical datasets or third party data, please ensure that the statement adheres to our policy

All data supporting the findings of this study are available within the paper, its supplementary information, and the supplied Source Data file. Further details on the study cohort-1 can be found in the ISRCTN registry (ISRCTN17974967, https://doi.org/10.1186/ISRCTN17974967). Source data are provided with this paper. Study protocols are available (in German language) upon request.
For the study we have used structural data from PDB: 6IW4, https://doi.org/10.2210/pdb6IW4/pdb

Research involving human participants, their data, or biological material

Policy information about studies with human participants or human data. See also policy information about sex, gender (identity/presentation), and sexual orientation and race, ethnicity and racism.

Reporting on sex and gender	The sex of study participants was determined through self-reported information. A total of 169 females and 81 males were recruited in Cohort 1. In Cohort 2 we had 12 females and 10 male participants. Gender was not specifically considered in the study design. In this manuscript we do not show any sex-based analyses. We did analyze the effect of sex on the reported outcomes in this manuscript; however, we did not find a significant effect of sex on the reported parameters/conclusions here presented. Additionally, the Source data file includes a column indicating the sex of the participant and the associated value for every result displayed in the figures. Sex information is disaggregated in the source data file.
Reporting on race, ethnicity, or other socially relevant groupings	Our cohorts recruited in an urban German environment were very homogenous concerning the self-reported race and ethnicity and these factors were not used further as confounding variates for the reported outcomes. All participants but three (in cohort-1) self-categorized themselves as caucasians.
Population characteristics	All the study participants were young healthy adults. 80.4% of the study cohort is 20-29 years old and 16.8% is 30-39 years old. In cohort 1 median age was 24 years (range 19-47), co-morbidities, illnesses or uncompatible pre-vaccinations resulted in exclusion from recruitment. In cohort 2 median age was 29 years (range 21-54).
Recruitment	--> For the study cohort 1 we recruited young healthy persons that planned to get the yellow Fever vaccine for traveling into risk areas at the outpatient clinic of the Division of Infectious Diseases and Tropical Medicine of the LMU university hospital. The study was advertised on the website of the Division of Infectious Diseases and Tropical Medicine of the LMU university hospital, in student chat groups and through postings on bulletin board of the university and hospital buildings. As a result, this recruitment is biased towards the inclusion of young medical students, predominantly females (67%), from an urban region. While these factors are not likely to significantly impact the results, it should be noted that the data presented here may not represent the elderly population.<br>--> Cohort 2 was recruited at the travel clinic of the microbiology Institut of the university of Erlangen. Participants attended the vaccination and travel consultation at the Erlangen University Hospital at the Microbiological Institute and got the yellow Fever vaccine for traveling into risk areas. There was no obvious selection bias that would affect the results.
Ethics oversight	Study participants of cohort 1 were recruited at the Division of Infectious Diseases and Tropical Medicine (DIDTM) as well as the Division of Clinical Pharmacology, University Hospital, LMU Munich, Germany. The study protocol was approved by the Institutional Review Board of the Medical Faculty of LMU Munich (IRB #86-16) and adhered to the most recent version of the declaration of Helsinki.<br>Study paricipants of cohort 2 were recruited at the Mikrobiologisches Institut of the University of Erlangen. The study protocol was approved by the ethics commitee of the Friedrich-Alexander-Universität Erlangen-Nürnberg (IRR AZ.350 20B) and adhered to the most recent version of the declaration of Helsinki. All donors gave informed consent.<br>For both cohorts, vaccinations were administered as standard-of-care consisting on 0.5ml Stamaril, Sanofi Pasteur.

Note that full information on the approval of the study protocol must also be provided in the manuscript.

Field-specific reporting

Please select the one below that is the best fit for your research. If you are not sure, read the appropriate sections before making your selection.

☒ Life sciences ☐ Behavioural & social sciences ☐ Ecological, evolutionary & environmental sciences

For a reference copy of the document with all sections, see nature.com/documents/nr-reporting-summary-flat.pdf

Life sciences study design

All studies must disclose on these points even when the disclosure is negative.

Sample size	The planned number of 250 participants was based on considerations regarding the variance of the parameters to be determined, which however in essential aspects for this study prior to the study start were unknown and could only be hypothesized. Because of this, and the exploratory nature of the study, a specific power analysis was not performed. Taking into account experiences on feasibility and the possibility of sample processing from a pilot study we estimated that a study of 200 -300 subjects will be necessary to detect meaningful differences based on host factors. The final sample size of the corresponding pre-vaccination status group reported in this manuscript was determined retrospectively using self-reported information and experimental data. Sample sizes for each measurement is shown in methods and figure legends. For cohort 2 22 participants were available for analysis.
Data exclusions	A total of 55 study participants were excluded from analysis due to discrepancies between self-reported vaccination status and confirmatory serological assays.
Replication	The data's reproducibility is ensured by the inclusion of a sufficient number of biological replicates (number of vaccinees). All assays were tested for consistency across multiple experimental days, with a minimum of 3 repetitions in pilot experimentation tests. However, limited sample availability did not allow for multiple repetitions for the same individual, type of assay, and sample. All the experiments included technical replicates. All assays utilized appropriate internal controls, defined in the methods section, to ensure inter-assay and inter-day comparability.
Randomization	Samples were not randomly allocated in experimental groups. Pre-vaccination status and post-vaccination timepoint were the two main variables considered for this study
Blinding	When not all the cohort samples were analysed, the experimentors beeing in person also the designer of the experiments stratified the cohort for futher analysis considering the two groups of interest. Therefore, blinding to group allocations was not possible.

Reporting for specific materials, systems and methods

We require information from authors about some types of materials, experimental systems and methods used in many studies. Here, indicate whether each material, system or method listed is relevant to your study. If you are not sure if a list item applies to your research, read the appropriate section before selecting a response.

Materials & experimental systems

n/a	Involved in the study	
☐	☒	Antibodies
☐	☒	Eukaryotic cell lines
☒	☐	Palaeontology and archaeology
☒	☐	Animals and other organisms
☐	☒	Clinical data
☒	☐	Dual use research of concern
☒	☐	Plants

Methods

n/a	Involved in the study	
☒	☐	ChIP-seq
☐	☒	Flow cytometry
☒	☐	MRI-based neuroimaging

Antibodies

Antibodies used	1) Anti-flavivirus 4G2, purified from hybridoma: D1-4G2-4-15 ATCC Cat# HB-112 with protein G purification<br>2) Anti-YFV 2D12, purified from hybridoma: 2D12 ATCC Cat# CRL-1689 with protein G purification<br>3) Anti-YFV 5A (Daffis et al 2005 and Lu et al 2019). Sequence is publicly available and the antibody was produced in mammalian expression system ExpiCHO (ThermoFisher)<br>4) E21.3 anti-YFV DII. Identified and produced in-house in mammalian expression system ExpiCHO (ThermoFisher)<br>5) Anti-human IgG HRP Jackson ImmunoResearch Cat# 109-035-088, dilution 1:5000<br>6) Anti-human IgM HRP ThermoFisher Cat# 31415, dilution 1:5000<br>7) Anti-human IgG Sigma Aldrich Cat# I2136, concentration 10 ug/ml<br>8) Fc Receptor Binding Inhibitor Polyclonal Antibody ThermoFisher Cat# 14-9161-73, concentration 10ug/ml<br>9) CD32 Antibody Bio-Rad Cat# MCA1075, concentration 10ug/ml<br>10) Anti-mouse-HRP cell signalling, 7076 dilution 1:1000
Validation	1) 4G2 antibody is a known FLE cross-reactive antibody (Crill JVirol 2004), which is commercially available. For this study, binding validation was performed in ELISA and SEC (Supplementary figure 6)<br>2) 2D12 antibody is yellow fever specific (Schlesinger virology 1983), commercially available. The exact binding site in DII is unknown. Binding valiation was done by ELISA.<br>3) 5A antibody produced in-house using the sequence published by (Daffis et al 2005). The exact binding site was published by Lu et al. CellRep 2019. In this study, binding validation was performed by ELISA and SEC (Supplemmentary figure 6). We have also confirmed the neutralizing capacity of this antibody.<br>4) E21.3 Produced in-house, known to bind domain II of YFV17D envelope protein at an undefined epitope in DII. Binding data are is shown in supplemetary figure 6 to all the E protein constructs used in this study<br>5) Anti-human IgG HRP. Polyclonal seconary antibody. Used according to manufacturer instructions for the ELISAs to quantify anti-YF

human IgG antibodies and 5A mAb.
6) Anti-human IgM HRP. Polyclonal. Used according to manufacturer instructions for the ELISA quantification of anti-YF IgM.
7) Anti-human IgG. Polyclonal. Used according to manufacturer instructions for coating the ELISpot plates for the detectection of IgG-secreting B cells with YF specificity.
8) Fc Receptor Binding Inhibitor. Polyclonal. Used according to manufacturer instructions and results shown in Figure 3A and Supplementary figure 2
9) CD32. Clone AT10 (Greenman et al. Mol Immunol 1991). Used according to manufacturer instructions and results shown in Figure 3A and Supplementary figure 2.
10) Anti-mouse-HRP cell signalling. Polyclonal. used for 4G2 detection in ELISA. (SF6)

Eukaryotic cell lines

Policy information about cell lines and Sex and Gender in Research

Cell line source(s)	PBMC of YF17D vaccinees. This study<br>drosophila S2 cell lines ThermoFisher Cat# R690-07<br>Vero B4 ATCC Cat# CCL81<br>THP-1 ATCC Cat# TIB-202<br>K562 ATCC Cat# CCL-243<br>A549 ATCC Cat# CCL-185
Authentication	None of the cells were authenticated
Mycoplasma contamination	All cells are mycoplasma negative. Cell cultures were routinely tested by PCR every 2-4 weeks
Commonly misidentified lines<br>(See ICLAC register)	The cell lines used here are not listed in the ICLAC.

Clinical data

Policy information about clinical studies

All manuscripts should comply with the ICMJE guidelines for publication of clinical research and a completed CONSORT checklist must be included with all submissions.

Clinical trial registration	The yellow fever vaccine cohort study was registered at the ISRCTN registry under the registration Nr. 17974967
Study protocol	We only have study protocolls in German, that were approved by the local IRB. These can be provided upon request but are not publically accessible.
Data collection	Study participants were recruited from 2015-2019 at the Division of Infectious Diseases and Tropical Medicine (DIDTM) as well as the Division of Clinical Pharmacology, University Hospital, LMU Munich, Germany and at the at the Mikrobiologisches Institut of the University of Erlangen in 2023 respectively.
Outcomes	Vaccine endpoints like antigen-specific antibody titers and neutralization were measured as principal indicators of vaccine-induced responses. Other experimental procedures were developed in a hypothesis-driven manner

Flow Cytometry

Plots

Confirm that:

- [x] The axis labels state the marker and fluorochrome used (e.g. CD4-FITC).
- [x] The axis scales are clearly visible. Include numbers along axes only for bottom left plot of group (a 'group' is an analysis of identical markers).
- [ ] All plots are contour plots with outliers or pseudocolor plots.
- [x] A numerical value for number of cells or percentage (with statistics) is provided.

Methodology

Sample preparation	Flow cytometry was used to determine virus infection using a venus encoding YF17D virus. After the infection cells were stained for viability and fixed in 4% PFA before acquisition of at least 10.000 live events.
Instrument	BD FACSCanto (sn: V96300312) and CytoFLEX LX (sn: BE48044)
Software	FlowJo, Diva
Cell population abundance	For infection measurement (neutralization and enhancement experiments) a total of 10.000 live cells were recorded per replicate

Gating strategy

FSC/SSC FSCH/FSCA SSC/Viability SSC/Infection (EGFP). Negative and positive gates were defined by uninfected control

☒ Tick this box to confirm that a figure exemplifying the gating strategy is provided in the Supplementary Information.
